# Field Borders Provide Winter Refuge for Beneficial Predators and Parasitoids: A Case Study on Organic Farms

**DOI:** 10.1093/jisesa/ieab027

**Published:** 2021-05-08

**Authors:** C Scott Clem, Alexandra N Harmon-Threatt

**Affiliations:** Department of Entomology, University of Illinois at Urbana-Champaign, 320 Morrill Hall, 505 S. Goodwin Ave., Urbana, IL 61801, USA

**Keywords:** natural enemy, field border, conservation biological control, semi-natural habitat, organic farming

## Abstract

Semi-natural field borders are frequently used in midwestern U.S. sustainable agriculture. These habitats are meant to help diversify otherwise monocultural landscapes and provision them with ecosystem services, including biological control. Predatory and parasitic arthropods (i.e., potential natural enemies) often flourish in these habitats and may move into crops to help control pests. However, detailed information on the capacity of semi-natural field borders for providing overwintering refuge for these arthropods is poorly understood. In this study, we used soil emergence tents to characterize potential natural enemy communities (i.e., predacious beetles, wasps, spiders, and other arthropods) overwintering in cultivated organic crop fields and adjacent field borders. We found a greater abundance, species richness, and unique community composition of predatory and parasitic arthropods in field borders compared to arable crop fields, which were generally poorly suited as overwintering habitat. Furthermore, potential natural enemies tended to be positively associated with forb cover and negatively associated with grass cover, suggesting that grassy field borders with less forb cover are less well-suited as winter refugia. These results demonstrate that semi-natural habitats like field borders may act as a source for many natural enemies on a year-to-year basis and are important for conserving arthropod diversity in agricultural landscapes.

The retention of semi-natural parcels in cultivated landscapes is a cornerstone of sustainable agriculture (Holland et al. 2016). These habitats are relatively undisturbed and permanent compared to arable fields and cover crops (Asbjornsen et al. 2013, Schulte et al. 2017), and increase landscape biological complexity (Fiedler et al. 2008, Asbjornsen et al. 2013, Hirsh et al. 2013, Pérez-Suárez et al. 2014, Werling et al. 2014, Schulte et al. 2017). Beneficial organisms like pollinators (Blaauw and Isaacs 2014, Werling et al. 2014, Schulte et al. 2017) and predators (Fiedler and Landis 2007, Werling et al. 2014, Blaauw and Isaacs 2015, Schulte et al. 2016, Schulte et al. 2017) find stable resources and shelter on these properties, allowing them to deliver their respective ecosystem services. The strategy of enhancing populations of naturally occurring predatory and parasitic arthropods (i.e., natural enemies) specifically is known as conservation biological control (Barbosa 1998, Fiedler et al. 2008, Holland et al. 2016, Gontijo 2019).

Some farming practices accommodate conservation biological control more than others. Organic farmers, for example, may use this to help compensate for the lack or reduction of synthetic insecticide input (Birkhofer et al. 2008). These growers are also required by the USDA to preserve semi-natural ‘buffer zones’ between them and conventional agriculture to prevent ‘co-mingling of organic and non-organic products’ (USDA AMS NOP 2011). In the midwestern United States, growers often restore these parcels as ‘pollinator-friendly’ wildflower strips resembling native perennial prairies (Hirsh et al. 2013, Schulte et al. 2017), a habitat of high conservation value (Samson and Knopf 1994). These field borders are thought to provide ecosystem services, but detailed information on the communities of natural enemies they support, particularly during winter months, is lacking.

While not all natural enemies such as predacious beetles, wasps, spiders, and other arthropods become inactive during winter, many diapause and emerge the following spring seeking prey (Pfiffner and Luka 2000, MacLeod et al. 2004). If natural enemies are not present at sufficient levels early in the season, they are less likely to manage pest problems before they breach economic threshold in spring and ensuing warm months (Dennis et al. 1994, Geiger et al. 2009). In many systems, the capacity of semi-natural habitats to support overwintering natural enemies is overlooked.

The primary objective of this study was to examine the spring emergence of potential natural enemies in field borders and adjacent organic cultivated crops in central Illinois. We compare abundance, species richness, and community dissimilarity of these arthropods in the two habitat types. Furthermore, we examine habitat variables to determine how they affect these arthropod communities.

## Methods

### Experimental Design

Five privately owned USDA certified organic fields with adjacent semi-natural field borders in central Illinois were examined. All sampled fields were under rotation but had soybean planted in them the previous fall. Soybean is a widely planted crop in the midwestern United States, including Illinois (USDA NASS 2017), and under organic conditions usually receives little to no insecticide input which may affect arthropod communities (McBride and Greene 2009). All field borders included grasses mixed with seeded and nonseeded forb species prevalent during the growing season. Only one site (Site 2) was not purposefully seeded with a native wildflower seed mix; this site had similar structural diversity to seeded sites.

**Fig. 1.**
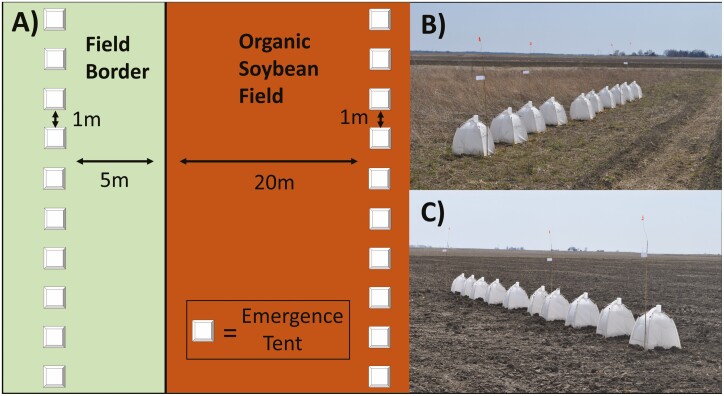
Tent arrangement at each site. (A) diagram of tent arrangements from an overhead point of view; (B–C) photographs of tents in semi-natural field border (B) and organic soybean field (C) at Site 3 in Danforth, IL. Photographs by CSC.

Arthropod overwintering density was measured using soil emergence tents (60 × 60 cm coverage, BugDorm BT2006) which were staked to the ground. Soil was packed onto the external flaps for stability and to prevent movement of insects into or out of tents. Trap bottles were filled with 350 ml of 20% propylene glycol, 80% water to euthanize and temporarily preserve specimens; surfactant (dish soap) was added to eliminate surface tension. Ten tents were placed 5 m into the border habitat at 1-m intervals parallel with the field, and ten were placed 20 m into the field at 1-m intervals parallel with the tents in the border (Fig. 1A–C). Tents were placed at sites on 14 March 2018 and samples were collected and placed into 70% ethanol on March 28, April 11, and April 25; the common practice of crop rotation at all sites impeded adequate multi-year replication. Seasonal conditions prior to March 28 in this region were not considered suitable for diapause termination in most arthropods. Predators (including omnivores) and parasitoids were extracted from samples and identified to species or morphospecies. Araneae (mostly immatures) and Aleocharinae staphylinid beetles were difficult to identify to a lower taxonomic unit, so they were pooled and treated as one group. For similar reasons, parasitoids were identified to the family level and then as morphospecies. All other specimens were identified to genus or species using appropriate dichotomous keys and taxonomic texts (see Supp Table S1 [online only] caption). Voucher specimens are deposited in the Illinois Natural History Survey Insect Collection (Dmitriev 2015, specimen identifiers 843590–843649, 843736–848405, 829421-829516).

Vegetation and ground cover estimates in both crop and field border were taken on April 11 and 25 April 2018 using 0.25 m^2^ quadrats. These were placed directly adjacent to each tent and used to estimate percent ground cover, forb cover (including seeded and nonseeded species), green grass cover, and dead plant material. Averages of these estimates were then used to assess effects on arthropod communities.

### Statistical Analyses

All statistical analyses were performed in R version 4.0.2 (R Core Team 2020). Generalized linear mixed-effects models (‘lme4’ package; Bates et al. 2015) were used to assess differences in overwintering arthropods between the two habitat types, specifying the distribution as poisson (with log link function), habitat (field vs border) and vegetation variables (grass and/or forb cover) as fixed effects, and tent nested within site (1|tent/site) and date (1|date) as random effects to correct for non-independence. Akaike information criterion (AICc) model selection (‘AICcmodavg’ package; Mazerolle 2020) was implemented to assess model fit and determine which habitat variables to include or exclude from the models. Percent bare ground and dead plant material were colinear with green grass cover (‘corrplot’ package; Wei and Simko 2017) and thus were excluded from all models. Two of the five crop fields were tilled, but including this potential effect in the models did not improve their fit. Species richness and abundance of overwintering predators, parasitoids, and predators + parasitoids were used as response variables. Aleocharine staphylinid beetles were excluded in the abundance analyses because their trophic level depends on species identity, which we were unable to determine. All models were inspected for overdispersion.

Nonmetric multidimensional scaling (NMDS) assessments followed by PerManova tests were used to assess differences in community composition between borders and fields with site as the replicate for an overall analysis via compiled data and tent as the replicate for site-level analysis. To assess which taxa contributed most to differences in community structure between these habitats for the overall assessment, we used the Bray-Curtis dissimilarity-based SIMPER analysis. NMDS, PerManova, and SIMPER analyses excluded Aleocharinae and were implemented using the ‘vegan’ package (Oksanen et al. 2019). The homogeneity of dispersion assumption for PerManova was confirmed to be satisfied using the PERMDISP2 procedure (betadisper function in ‘vegan’).

## Results

**Fig. 2.**
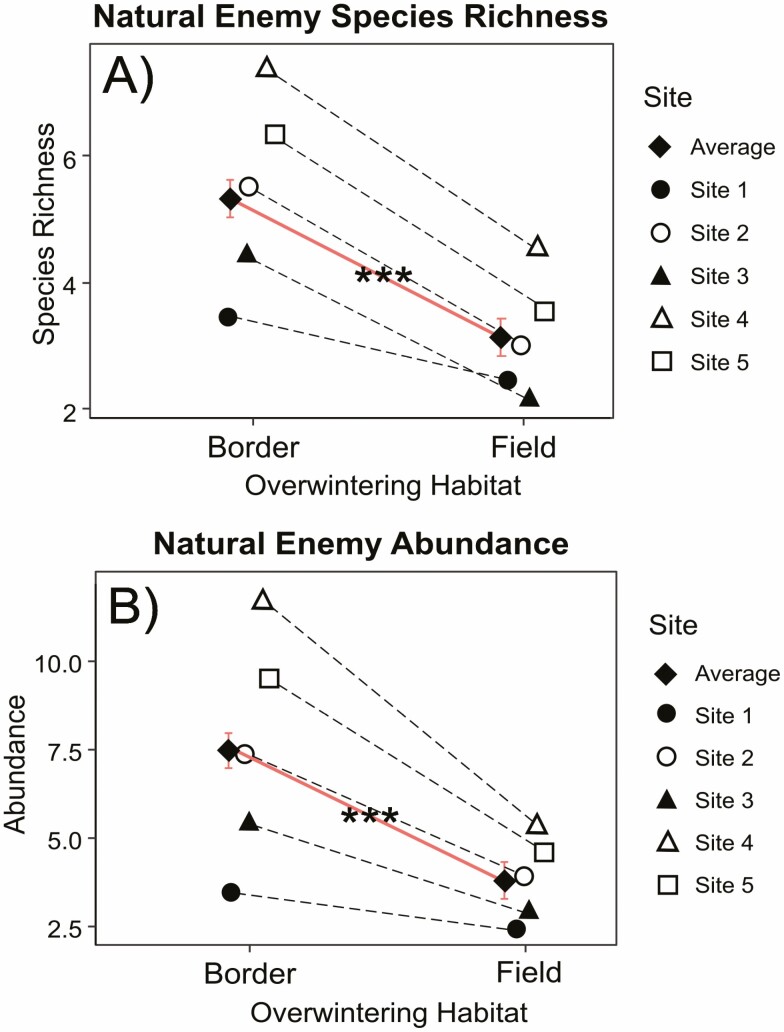
Average species/morphospecies richness (A) and abundance (B) of natural enemy species per tent compared between semi-natural field borders and cultivated organic fields (Wald Z-test, error bars = standard error). Site values are the total numbers pooled across each habitat. Test statistics for (A) Z = −5.537, *P* < 0.001***; (B) Z = −11.869, *P* < 0.001***.

In total, 4,226 potential natural enemies were collected and identified to 95 species or morphospecies of predatory (42 sp.) or parasitic (53 morphsp.) arthropods (Supp Table S1 [online only]). Field borders hosted more species (Z = −5.440, *P* < 0.001***) and a greater abundance (Z = −11.869, *P* < 0.001***) of overwintering predators and parasitoids than did cultivated fields (Table 1, Fig. 2). Species richness and abundance were generally positively associated with percent forb cover and negatively associated with grass cover (Table 1).

**Table 1.** Results of generalized linear mixed-effects models comparing abundance and species richness of predators and parasitoids (potential natural enemies) overwintering at five sites, specifying the distribution as poisson (with log link function), habitat (field vs. border) and vegetation variables (grass and/or forb cover) as fixed effects, and tent nested within site (1|tent/site) and date (1|date) as random effects (‘lme4’ package; Bates et al. 2015)

Potential natural enemy species richness						
Arthropod group	Explanatory variable	Estimate	Standard error	Df residuals	Z value (Wald statistic)	Pr(>|z|)
Predators	(Intercept)	1.079	0.414	294	2.607	0.009**
	Habitat Field	−0.478	0.091		−5.271	<0.001***
	Forb Cover	0.003	0.003		0.8	0.424
Parasitoids	(Intercept)	−0.503	0.702	294	−0.717	0.473
	Habitat Field	−0.275	0.156		−1.763	0.078
	Forb Cover	0.015	0.005		2.742	0.006**
Predators + parasitoids	(Intercept)	1.284	0.461	294	2.784	0.005**
	Habitat Field	−0.436	0.079		−5.537	<0.001***
	Forb Cover	0.005	0.003		1.881	0.06 .
Potential natural enemy abundance						
Arthropod group	Explanatory variable	Estimate	Standard error	Df residuals	Z value (Wald statistic)	Pr(>|z|)
Predators (excluding Aleocharinae)	(Intercept)	1.19	0.578	295	2.061	0.039*
	Habitat Field	−0.82	0.063		−12.965	<0.001***
Parasitoids	(Intercept)	0.358	0.814	294	0.44	0.66
	Habitat Field	−0.631	0.112		−5.618	<0.001***
	Green Grass	−0.018	0.005		−3.678	<0.001***
Predators + parasitoids (excluding Aleocharinae)	(Intercept)	1.652	0.597	294	2.769	0.006**
	Habitat Field	−0.773	0.065		−11.869	<0.001***
	Green Grass	−0.008	0.003		−2.467	0.014*

**Fig. 3.**
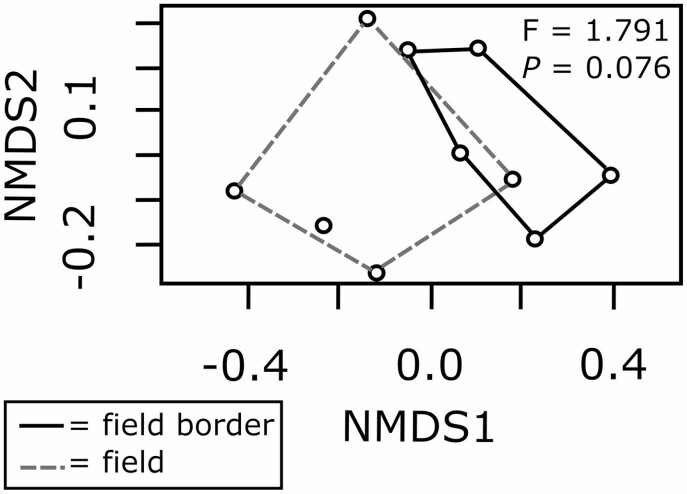
Nonmetric multidimensional scaling (NMDS) plots and PerManova analyses (df = 1,9) of overwintering natural enemy communities compiled by site in cultivated fields and field borders. Points represent sites.

There was a trend that overall community composition of overwintering natural enemies were distinct between fields and field borders (*F*_1,4_ = 1.79, *P* = 0.07; Fig. 3). For individual site-level analyses, this trend was significant at all sites except Site 1 (*F*_1, 19_ = 1.24, *P* = 0.29; Supp Fig. S1). Results of the SIMPER analysis revealed the top three dissimilarity-contributing taxa to be spiders (Araneae; 10.47%), a morphospecies of eucoiline parasitoid wasp (Hymenoptera: Figitidae; 8.85%), and *Oxytelus* Gravenhorst 1802 beetles (Coleoptera: Staphylinidae; 8.24%). Of the eight natural enemy taxa that contributed more than 5% dissimilarity, six were more prevalent in borders while two were more prevalent in cultivated fields (Table 2).

**Table 2.** SIMPER analysis of differences in overwintering natural enemy community composition between field borders and organic cultivated fields. Taxa listed in this table contributed more than 5% of the dissimilarity

Taxon	Order	Family	Preferred habitat	Mean abundance in preferred habitat	Difference in mean abundance	Percent dissimilarity contributed
Araneae	Araneae	-	Border	29.2	19	10.47
Eucoilinae sp. 1	Hymenoptera	Figitidae	Field	20.8	13.2	8.85
*Oxytelus* Gravenhorst 1802	Coleoptera	Staphylinidae	Border	20.8	16.8	8.24
*Agonoleptus conjunctus* (Say, 1823)	Coleoptera	Carabidae	Border	22.8	9.6	7.53
*Stenolophus comma* (Fabricius, 1775)	Coleoptera	Carabidae	Field	16.2	7.4	6.88
Megaspilidae sp. 1	Hymenoptera	Megaspilidae	Border	19.4	9.8	6.66
*Bradycellus rupestris* (Say, 1823)	Coleoptera	Carabidae	Border	16	8	6.5
*Olophrum obtectum* Erichson, 1840	Coleoptera	Staphylinidae	Border	15.4	11.2	5.47

## Discussion

Our study provides important, detailed information on the capacity of uncultivated habitat to offer overwintering refugia for potential natural enemies. Field borders supported a greater abundance and species richness of these arthropods compared to cultivated organic crop fields (Table 1, Fig. 2). Furthermore, there was a trend for distinct community composition between the two habitat types (Fig. 3 and Supp Fig. S1 [online only]), with many species found in border habitat that were either undetected or rarely detected in cultivated fields (Supp Table S1 [online only]). These data demonstrate that uncultivated field borders may play a key role as a source for natural enemy biodiversity at the beginning of each growing season in simplified midwestern agricultural landscapes.

Differences in community composition between the two habitat types (cultivated field vs field border) were evident at four sites, with site one being an outlier (Supp Fig. S1 [online only]). The field border at this site was mowed the previous fall and consisted of mostly grasses and few forbs, resulting in relatively simplified habitat. This is evident in our findings that arthropod species richness and abundance were negatively associated with grass cover and positively associated with forb cover (Table 1) and suggests that field border complexity and diversity is an important predictor of natural enemy diversity. Of the eight species that drive dissimilarity between the two habitats, only a morphospecies of eucoiline wasp (Hymenoptera) and the carabid *Stenolophus comma* (Fabricius 1775) (Coleoptera) were found overwintering more prevalently in the cultivated fields (Table 2). Eucoiline wasps are koinobiont endoparasitoids of various cyclorrhaphous Diptera (Gallardo et al. 2017) which may have also been prevalent in fields, and *Stenolophus comma* is an omnivorous seedcorn beetle common in midwestern agricultural landscapes that may occur in field habitats due to the prevalence of seeds (Capinera 2001).

Maintaining biodiversity of predatory and parasitic arthropods as potential natural enemies can strengthen the resiliency of agroecosystems to pests (van Alebeek et al. 2006, Gardiner et al. 2009, Geiger et al. 2009, Chaplin-Kramer 2011, Woltz et al. 2012, Blaauw and Isaacs 2015, Jonsson et al. 2017, Gontijo 2019), and our findings demonstrate that semi-natural field borders can benefit this biodiversity by providing overwintering refugia. In midwestern organic agriculture, an early season presence of these arthropods may lessen impacts of major pests of corn, soybean, wheat, and other common crops, although this should be tested. Different natural enemy taxa can work in tandem, attacking pests through diverse modes of action, in dissimilar micro-habitats, and at separate life-stages (Jonsson et al. 2017, Snyder 2019). While taxa identified in our study like many carabids and staphylinids (Bousquet 2010, Betz et al. 2018) are opportunistic feeders or omnivores that prey upon both pestiferous or non-pestiferous species, their presence is important. Maintaining multiple species encompassing similar niches (i.e., functional redundancy) or with the ability to switch food resources acts as insurance for when dominant species suffer population loss, allowing less-dominant species to take their place (Jonsson et al. 2017). Some may incidentally provide added benefits, like some species in the carabid subfamily Harpalinae which devote much of their diet to weed seeds thus offering weed suppression services (Menalled et al. 2007, Kulkarni et al. 2015). Landscapes that are depauperate of semi-natural habitats are less likely to include this diversity.

Maintaining semi-natural habitat on properties is a simple method for reducing agricultural impacts on the environment and enhancing biodiversity and ecosystem services. With help from programs like the USDA Conservation Reserve Program, these habitats may be improved and modified for specific conservation purposes at relatively low cost (Harris and Iyer 2014), which may further improve overwintering arthropod diversity. Our results provide evidence that these properties, particularly those which are complex and diverse, can offer refugia for a plethora of overwintering pest natural enemies. In absence of these types of habitat, natural enemies are less likely to be prevalent early in the season, which can be critical for suppressing pests before they become problematic (Dennis et al. 1994, Geiger et al. 2009). In order to fully assess the role of semi-natural habitats in sustainable pest management, it is crucial that we understand their capacity to offer winter refugia for natural enemies.

## Supplementary Material

ieab027_suppl_Supplementary_Materials

## Data Availability

Raw data and code is available for viewing on the Illinois Data Bank (https://doi.org/10.13012/B2IDB-8470827_V1).
