# Supplementary material for: Field Borders Provide Winter Refuge for Beneficial Predators and Parasitoids: A Case Study on Organic Farms
Source: J Insect Sci. 2021 May 8;21(3):2. doi: 10.1093/jisesa/ieab027 (PMC8106474; doi:10.1093/jisesa/ieab027)
Supplement: ieab027_suppl_Supplementary_Materials [file ieab027_suppl_supplementary_materials.docx]

| **Order** | **Family** | **Species or morphospecies** | **Total in field boundary** | **Total in cultivated fields** |
| --- | --- | --- | --- | --- |
| **Predators/omnivores** | | | | |
| Araneae |  |  | 146 | 51 |
| Coleoptera Total | | | 1,520 | 1,883 |
| Coleoptera Total excluding Aleocharinae | | | 587 | 277 |
| Coleoptera | Carabidae | Total | 320 | 208 |
|  |  | *Amara* sp*.* Bonelli, 1810 | 8 | 0 |
|  |  | *Agonoleptus conjunctus*  (Say, 1823) | 114 | 66 |
|  |  | *Agonum punctiforme* (Say, 1823) | 4 | 0 |
|  |  | *Anisodactylus sanctaecrucis*  (Fabricius, 1798) | 11 | 3 |
|  |  | *Bembidion* Latreille, 1802 sp*.* 1 | 14 | 6 |
|  |  | *Bembidion* Latreille, 1802 sp*.* 2 | 3 | 0 |
|  |  | *Bradycellus atrimedeus* (Say, 1823) | 1 | 0 |
|  |  | *Bradycellus badipennis* (Haldeman, 1843) | 9 | 1 |
|  |  | *Bradycellus rupestris* (Say, 1823) | 80 | 40 |
|  |  | *Harpalus herbivagus*  Say, 1823 | 14 | 7 |
|  |  | *Harpalus* Latreille 1802 sp*.* 2 | 2 | 0 |
|  |  | *Poecilus chalcites*  (Say, 1823) | 1 | 1 |
|  |  | *Pterostichus* sp.  Bonelli, 1810 | 0 | 1 |
|  |  | *Stenolophus comma* (Fabricius, 1775) | 44 | 81 |
|  |  | *Stenolophus fuliginosus* Dejean, 1829 | 2 | 1 |
|  |  | *Stenolophus ochropezus* (Say, 1823) | 14 | 1 |
| Coleoptera | Coccinellidae | Total | 25 | 4 |
|  |  | *Coleomegilla maculata* De Geer, 1775 | 2 | 0 |
|  |  | *Cycloneda munda*  (Say, 1835) | 1 | 0 |
|  |  | *Diomus* sp.  Mulsant, 1850 | 25 | 6 |
|  |  | *Hippodamia variegata* (Goeze, 1777) | 1 | 0 |
| Coleoptera | Staphylinidae | Total | 1,175 | 1,671 |
|  |  | Aleocharinae sp. | 936 | 1,609 |
|  |  | Staphylinini sp. | 2 | 3 |
|  |  | *Homaeotarsus* sp.  Hochhuth, 1851 | 1 | 0 |
|  |  | *Lathrobium* sp. Gravenhorst, 1802 | 1 | 0 |
|  |  | *Mycetoporus lucidulus* LeConte, 1863 | 2 | 4 |
|  |  | *Nudobius cephalus*  (Say, 1834) | 4 | 3 |
|  |  | *Olophrum obtectum* Erichson, 1840 | 77 | 21 |
|  |  | *Oxytelus* sp.  Gravenhorst, 1802 | 104 | 20 |
|  |  | *Philonthus* sp*.* 1  Stephens, 1829 | 10 | 3 |
|  |  | *Philonthus* sp. 2  Stephens, 1829 *Philonthus* sp*.* 3 Stephens, 1829  *Stenus* sp. Latreille, 1797 | 1  0  5 | 1  1  0 |
|  |  | *Stenistoderus* sp. Jacquelin du Val, 1856 | 0 | 1 |
|  |  | *Tachyporus nitidulus* (Fabricius, 1781) | 26 | 7 |
|  |  | *Tachyporus* sp. 2 Gravenhorst, 1802 | 9 | 1 |
| Diptera | Syrphidae | *Eupeodes americanus* (Wiedemann 1830) | 0 | 1 |
| Hemiptera | Anthocoridae  Nabidae | *Orius insidiosus*  (Say, 1832)  *Nabis roseipennis*  Reuter, 1872 | 7  37 | 12  6 |
| Hymenoptera | Formicidae | Total | 27 | 9 |
|  |  | *Lasius americanus*  Emery, 1893 | 25 | 5 |
|  |  | *Myrmica* sp.  Latreille, 1804 | 1 | 0 |
|  |  | *Tapinoma sessile*  (Say, 1836) | 0 | 1 |
| Neuroptera | Hemerobiidae | *Hemerobius stigma* Stephens, 1835 | 0 | 1 |
| **Parasitoids** | | | | |
| Hymenoptera | | | 312 | 212 |
|  | Aphelinidae | 2 morphospecies | 6 | 2 |
|  | Bethylidae | 1 morphospecies | 0 | 1 |
|  | Braconidae | 2 morphospecies | 8 | 3 |
|  | Ceraphronidae | 1 morphospecies | 4 | 2 |
|  | Diapriidae | 4 morphospecies | 62 | 11 |
|  | Encyrtidae | 2 morphospecies | 3 | 0 |
|  | Figitidae: Eucoilinae | 3 morphospecies | 40 | 106 |
|  | Eulophidae | 3 morphospecies | 3 | 2 |
|  | Eupelmidae | 1 morphospecies | 0 | 1 |
|  | Ichneumonidae | 6 morphospecies | 9 | 7 |
|  | Megaspilidae | 2 morphospecies | 97 | 49 |
|  | Mymaridae | 3 morphospecies | 8 | 3 |
|  | Platygastridae | 1 morphospecies | 2 | 1 |
|  | Pteromalidae | 8 morphospecies | 25 | 5 |
|  | Scelionidae | 11 morphospecies | 41 | 21 |
|  | Tetracampidae | 1 morphospecies | 1 | 0 |
|  | Trichogrammatidae | 1 morphospecies | 6 | 1 |
| Diptera | Tachinidae | 1 morphospecies | 2 | 0 |
| **Total arthropods excluding Aleocharinae** | | | **972** | **518** |
| **Total arthropods** | | | **2,051** | **2,175** |

**Supplementary Table S1:** list of species and morphospecies of pest natural enemies overwintering in wildflower-strip field borders and cultivated organic fields via emergence tents. Taxonomic resources used for identifications are as follows: Carabidae: Arnett and Thomas 2001, Bousquet 2010, Bousquet 2012, Evans 2014; Coccinellidae: Evans 2014; Staphylinindae: Campbell 1979, Campbell 1983, Campbell 1991, Arnett and Thomas 2001; Syrphidae: Skevington and Locke 2019; Anthocoridae: Kelton 1978; Nabidae: Swanson 2012; Formicidae: AntWeb 2019; Hemerobiidae: Carpenter 1940; Parasitoids: Goulet and Huber 1993.

**References**

**AntWeb**. **2019**. AntWeb, version 8.44. California Academy of Science. <https://www.antweb.org>

**Arnett, R. H. Jr, and M. C. Thomas. 2001**. American Beetles, Volume I: Archostemata, Myxophaga, Adephaga, Polyphaga: Staphyliniformia. CRC Press LLC, Boca Raton, FL.

**Bousquet, Y. 2012**. Catalogue of Geadephaga (Coleoptera, Adephaga) of America, north of Mexico. Zookeys 245: 1–1722.

**Campbell, J. M. 1979**. A revision of the genus Tachyporus Gravenhorst (Coleoptera: Staphylinidae) of North America and Central America. Mem. Ent. Soc. Can. 109: 95.

**Campbell, J. M. 1983**. A revision of the North American Omaliinae (Coleoptera: Staphylinidae). 4. The genus Olophrum Erichson. Can. Entomol. 115: 577–622.

**Campbell, J. M. 1991**. A revision of the genera Mycetoporus Mannerheim and Ischnosoma Stephens (Coleoptera: Staphylinidae: Tachyporinae) of North and Central America. Mem. Ent. Soc. Can. 156: 1–169.

**Carpenter, F. M. 1940**. A revision of the Nearctic Hemerobiidae, Berothidae. Sisyridae, Polystoechotidae and Dilaridae. P. Am. Acad. Arts Sci. 74: 193–280.

**Evans, A. V. 2014**. Beetles of Eastern North America. Princeton University Press, Princeton, NJ.

**Goulet, H., and J. T. Huber. 1993**. Hymenoptera of the world: An identification guide to families. Agriculture Canada, Ottawa, Ontario.

**Kelton, L. A. 1978**. The Insects and Arachnids of Canada Part 4: The Anthocoridae of Canada and Alaska. Supply and Services Canada, Ottawa, Ontario.

**Skevington, J. H., and M. M. Locke. 2019**. Field Guide to the Flower Flies of Northeastern North America. Princeton University Press, Princeton, NJ.

**Swanson, D. R. 2012**. A synopsis of the damsel bugs (Heteroptera: Nabidae) of Michigan. Great Lakes Entomol. 45: 4.4

**
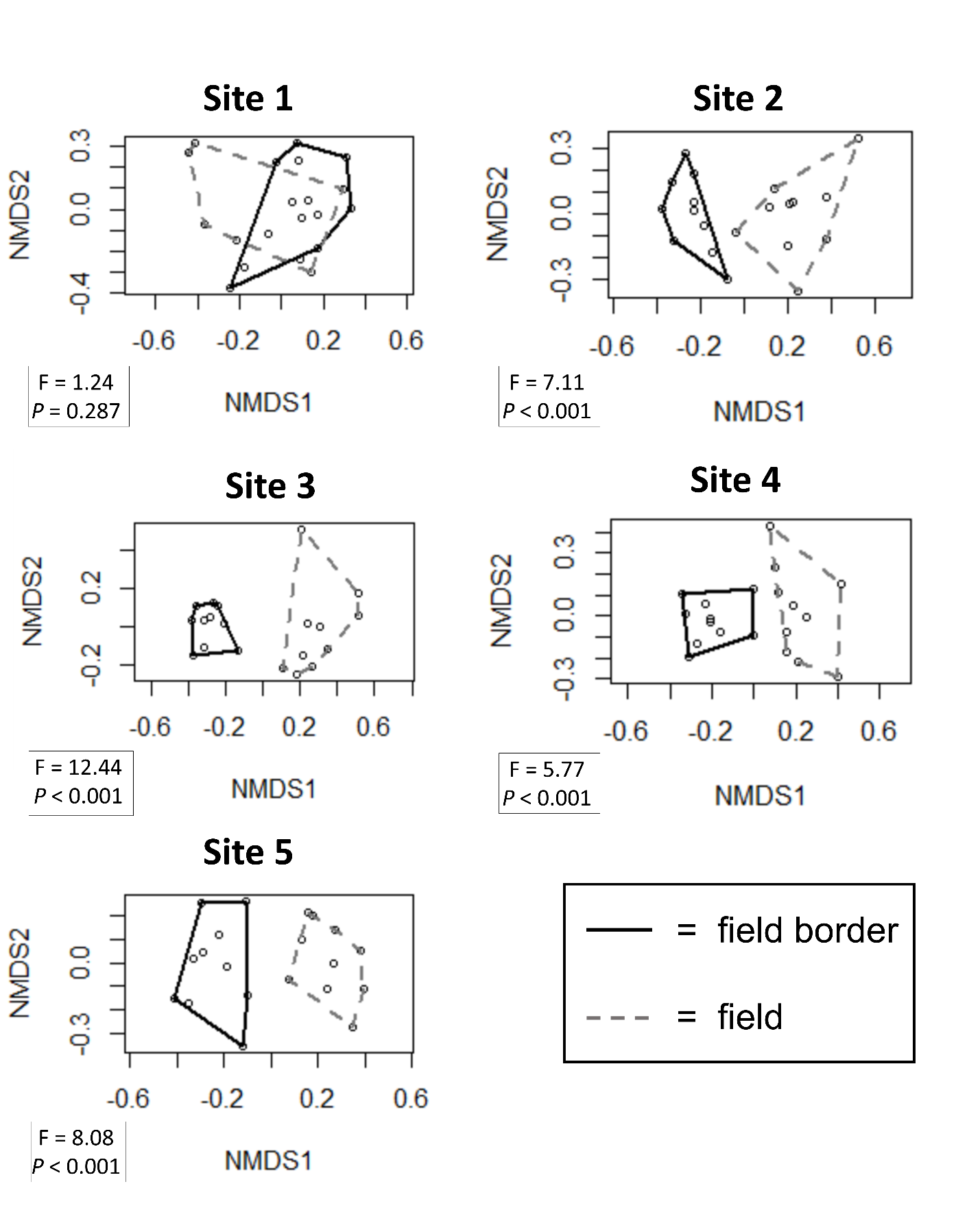
**

**Supplemental Figure S1:** Non-metric multidimensional scaling (NMDS) plots and PerManova analyses (df = 1,19) of overwintering natural enemy communities in wildflower-strip field borders and cultivated organic fields. Points represent tents.
